# Diffusion MRI tractography of the human heart *In Vivo* at end-diastole and end-systole

**DOI:** 10.1186/1532-429X-14-S1-O49

**Published:** 2012-02-01

**Authors:** Choukri Mekkaoui, Sonia Nielles-Vallespin, Peter D Gatehouse, Marcel P Jackowski, David N Firmin, David E Sosnovik

**Affiliations:** 1 Harvard Medical School-MGH-Athinoula A. Martinos Center for Biomedical Imaging, Charlestown, MA, USA; 2 Radiology, Massachusetts General Hospital, Harvard Medical School, Boston, MA, USA; 3 CMR Unit, Royal Brompton Hospital, London, UK; 4 Computer Science, University of São Paulo, Institute of Mathematics and Statistics, São Paulo, Brazil; 5 Cardiology Division, Massachusetts General Hospital, Harvard Medical School, Boston, MA, USA

## Summary

Diffusion Tensor MRI (DTI) of the human heart *in vivo* has to date been performed in 2D and at a single phase of the cardiac cycle. Here we perform 3D tractography of the human heart *in vivo* at both end diastole and end systole. We show that fiber orientation in the subepicardium becomes more oblique during systole, and that scalar indices of diffusion (mean diffusivity and fractional anisotropy) decrease during systole. Our data suggest that myocardial fiber architecture is dynamic and is a function of both chamber geometry and myocardial contraction.

## Background

Diffusion Tensor Imaging (DTI) of the human heart *in vivo* has been described [1,2] but has only been performed in 2D and at a single phase of the cardiac cycle. The impact of myocardial contraction on 3D fiber architecture *in vivo* thus remains poorly defined. Here we use a recently developed diffusion-weighted stimulated echo single shot EPI sequence [3], to address this question with a 3D tractographic approach [4]. The purpose of this study was thus to perform *in vivo* DTI of normal human hearts at end-diastole and end-systole and quantify changes in myofiber organization as the myocardium contracts and relaxes.

## Methods

DTI of three normal volunteers was performed on a 3T scanner (Skyra, Siemens) with the following parameters: 6 diffusion-encoding directions, b=350s/mm^2^, TR/TE=1100/23ms, BW=2442Hz/pixel, spatial resolution=2.7x2.7x8mm^3^, 3 slices, 6-8 averages, multiple breathholds. Fiber tracts were constructed by integrating the primary eigenvector field from the dyadic tensor into streamlines using a 4th order Runge-Kutta approach. Myofiber tracts were color-coded by their median helix angle [4]. Mean diffusivity (MD), fractional anisotropy (FA) and individual eigenvalue maps were averaged in 12 sectors of the anterior, lateral, inferior and septal walls of the left ventricle (LV).

## Results

**Figure 1:**
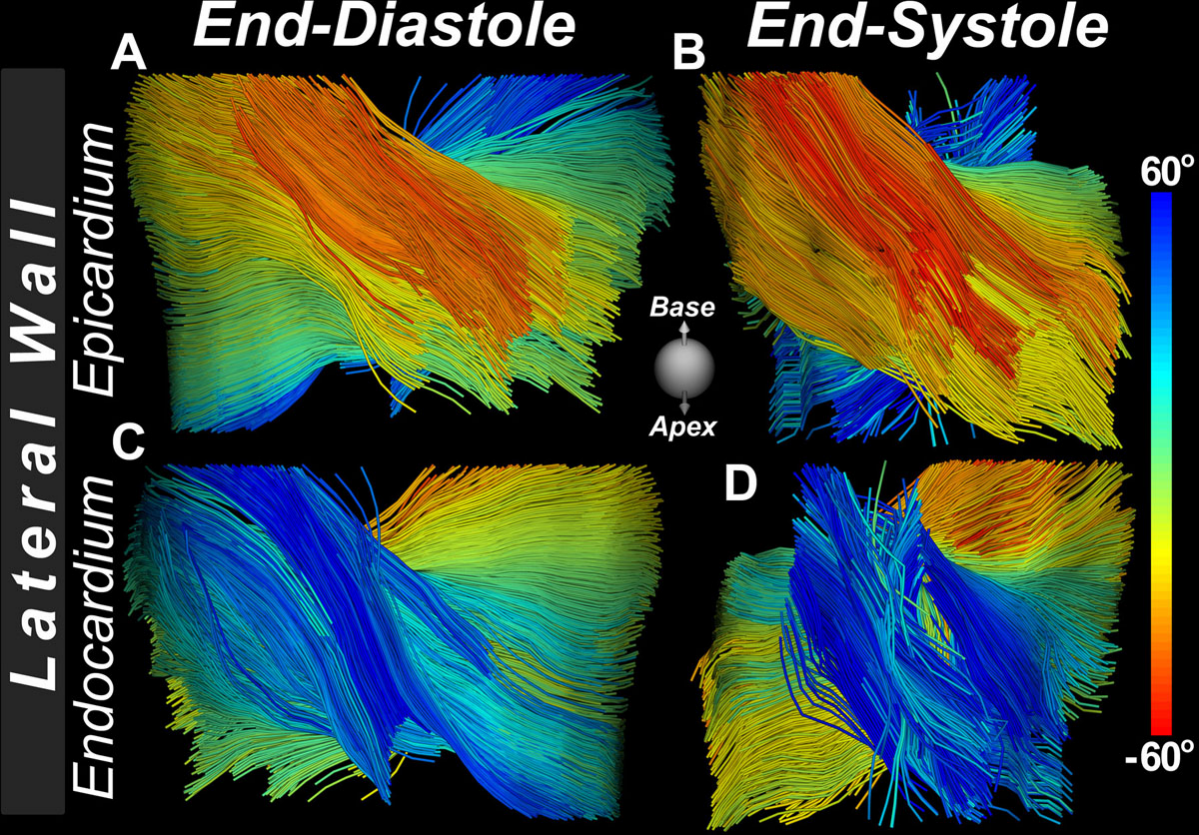
DTI tractography of the heart *in vivo* at end-diastole (**A, C**) and end-systole (**B, D**). Fiber tracts are viewed from their subepicardial (**A, B**) and subendocardial surfaces (**C, D**) and are color-coded by their median helix angle. Fiber tracts in the subepicardium at end-systole become more oblique (red) as the myocardium contracts. However, at end-diastole, as the LV outer circumference expands, the subepicardial fibers assume a more right-handed (less oblique) orientation.

**Figure 2:**
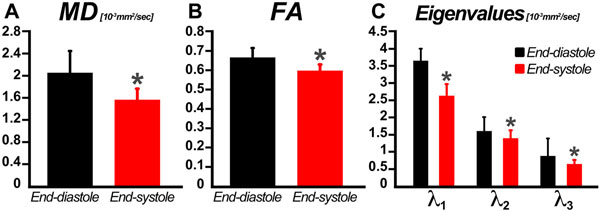
Bar plots of MD, FA and diffusion tensor eigenvalues computed across all 12 sectors within the LV of 3 normal human hearts. As the myocardium contracts (from end-diastole to end-systole), extracellular volume decreases and the myocytes thicken, resulting in concomitantly reduced MD and FA. The reduction in diffusivity and anisotropy is confirmed by a proportional decrease in all three eigenvalues.

Tractography showed that myofibers in the subepicardium of the LV assumed a more oblique orientation at end-systole *versus* end-diastole (Figure 1). MD and FA were significantly (p<0.05, Mann-Whitney) higher at end-diastole than end-systole (Figure 2). Likewise, all three eigenvalues were higher at end-diastole than end-systole (p<0.05), although the change in the principal eigenvalues (λ1) was greatest.

## Conclusions

Here, for the first time, we perform DTI tractography of the human heart *in vivo* at two phases of the cardiac cycle. The decrease in MD and FA at end-systole is likely due to myocyte thickening and compression of the extracellular space during systole. The increase in fiber obliquity in the subepicardium in systole could play an important role in base-apex shortening. Further study will be needed to determine the relationship between myocardial strain and the diffusion tensor. However, our data suggest that myofiber architecture in the human heart is dynamic and is a function of both chamber geometry and LV contraction.

## Funding

R01 HL093038 (Sosnovik), NCRR P41RR14075 (Martinos Center) and MGH-ECOR (Mekkaoui).
